# Enhancing Fatigue Resistance in Asphalt Mixtures with a Novel Additive Derived from Recycled Polymeric Fibers from End-of-Life Tyres (ELTs)

**DOI:** 10.3390/polym16030385

**Published:** 2024-01-30

**Authors:** Gonzalo Valdes-Vidal, Alejandra Calabi-Floody, Cristian Mignolet-Garrido, Cristobal Bravo-Espinoza

**Affiliations:** 1 Department of Civil Engineering, Universidad de La Frontera, Temuco 4811230, Chile; alejandra.calabi@ufrontera.cl (A.C.-F.); cristian.mignolet@ufrontera.cl (C.M.-G.); 2 GiPAV—Grupo de Investigación en Pavimentación Vial, Temuco 4811230, Chile; ing.cristobalbravo@gmail.com

**Keywords:** end-of-life-tires (ELT), ELT-based additive (WTTF), hot-mix asphalt (HMA), stone mastic asphalt (SMA), fatigue properties, stiffness, dissipated energy

## Abstract

Waste-tire textile fibers (WTTF) represent a challenge for the recycling industry since there are currently very few alternatives for their use. In this study, an evaluation of the effect of a new additive developed in two granular formats from WTTF on the fatigue behavior of asphalt mixtures was performed. For the first format of the WTTF-based additive, its effect was evaluated on hot-mix asphalt (HMA), while for the second format of the additive, the effects were evaluated on stone mastic asphalt (SMA). This second format represents an alternative that allows for the total replacement of the cellulose stabilizing additive used in the reference mix. The evaluation of fatigue damage in the mixes was performed using the four-point bending beam (4PB) test specified in European standard EN 12697-24. The test results show that the asphalt mixtures manufactured with WTTF-based additives exhibited a higher capacity to resist load cycles before failure compared to the reference mixtures. Likewise, once the asphalt mixtures were evaluated in a pavement structure by means of an empirical mechanistic analysis, the pavement structures composed of asphalt mixtures with WTTF-based additives showed significant improvements in their durability for the different load axes evaluated. For an average thickness of 15 cm of asphalt mix of a pavement-type structure, the use of the WTTF additive increases the durability of the structures by up to 129% and 112% compared to the HMA and SMA reference mixtures, respectively. These results show that both formats of the WTTF-based admixture improve the fatigue damage resistance of the HMA and SMA asphalt mixtures.

## 1. Introduction

Worldwide, the annual generation of end-of-life tires (ELTs) exceeds 1.5 billion units [1,2], which poses a worrying situation environmentally [3]. This constant increase in ELTs is directly related to the continuous development of the automotive sector and the growing demand for tires [4]. In Europe, about 324 million new tires were imported in 2020, of which approximately 89.5% were used in passenger cars, 4.9% in heavy vehicles, and the remaining 5.5% in other types of transport vehicles [5]. Once these tires reach end-of-life, most of them are discarded and deposited in landfills, which has generated an accumulation of about 4 billion ELTs worldwide [6]. These figures show the magnitude of the problem and emphasize the need to implement measures for the management and treatment of these wastes.

Three main components can be obtained from the recycling of ELTs: rubber (45–70% by weight); steel fibers (5–30% by weight); and polymeric fibers (5–15% by weight) [7,8,9,10,11]. These components are separated by size reduction processes, which facilitates their subsequent recycling and reuse [12]. Recycled rubber and steel fibers from tires are widely used by the recycling industry in various applications [13,14,15,16,17,18,19,20,21]. However, polymeric fibers from ELTs (WTTF) present challenges in their industrial application due to their limited use. In most cases, these fibers either end up in landfills or are used as combustion material in cement plants, which generates pollutant gas emissions [11]. According to the literature, WTTFs are mainly composed of polyester and polyamide polymers, such as nylon 6 or nylon 6.6 [8,22,23].

Asphalt mixes play a fundamental role in the construction of flexible pavements. Among these, hot-mix asphalt (HMA) is the most widely used [24,25], with a total production of 290.6 million tons in Europe and 392.0 million tons in the United States in 2021 [26]. However, in situations of heavy traffic or extreme environmental conditions, high-performance mixes are required, such as special mixes of the stone mastic asphalt (SMA) type [27,28]. SMA mixes are characterized by their high strength and high asphalt content, which requires the use of stabilizing or inhibiting additives to prevent runoff of the asphalt binder [29,30].

Asphalt mixtures are viscoelastic materials by nature, which implies that they possess both viscous and elastic properties [31,32,33]. In a viscoelastic state, the pavement has the ability to dissipate stresses through deformation. The viscous part of the material allows for the pavement to deform temporarily, absorbing stresses and avoiding stress concentration at specific points [33]. Meanwhile, the elastic part of the material causes the pavement to recover part of its original shape once the load is removed [33]. However, with the passage of time, the accumulated deformation can lead to crack formation and fatigue damage [34]. Fatigue cracking is a critical phenomenon that can cause premature failure of flexible pavements, significantly impacting their serviceability, structural strength, and appearance [34]. Possible causes of fatigue cracking include poor pavement design, repeated traffic loads exceeding accessible limits, and inappropriate choice of construction materials [35]. Therefore, it is critical to understand and address this phenomenon to ensure pavement durability.

In this context, the incorporation of fiber in asphalt pavements has been widely used and studied, concluding that its addition generates important benefits in the mechanical behavior of asphalt mixtures; however, Wu et al. (2023) indicate that the type of fiber and its characteristics determine important properties that are fundamental for the effectiveness of its incorporation into asphalt [36]. Numerous studies have conclusively demonstrated that synthetic fibers can be used effectively to improve the performance of asphalt mixtures. A review by Guo et al. (2023) [37] and Jia et al. (2023) [38] concluded that fibers are an excellent reinforcement additive, as they contribute to improving the mechanical performance of asphalt mixtures. These fibers provide benefits in terms of resistance to permanent deformation, water sensitivity, tensile strength, fatigue cracking resistance, and overall durability of the asphalt mixture. These findings are consistent with previous studies by Wu et al. (2008) [39] and Ye et al. (2009) [40], who found that synthetic fibers, such as polyester, improve the cracking resistance and extend the fatigue life of asphalt mixtures. In another study, Yin and Wu (2018) [41] evaluated the effects of nylon addition to SMA mixtures, observing improvements in mixture stability at high temperatures, resistance to cracking at low temperatures, and moisture damage. On the other hand, Zhang et al. (2020) [42] studied an open-graded friction course (OGFC) asphalt mixture and showed that the use of polyester fibers improved drainage properties, resistance to permanent deformation, and doubled fatigue cracking resistance. These results align with other studies that have researched the benefits of using WTTF on the mechanical performance of asphalt mixtures. Calabi et al. (2022) [43] and Calabi et al. (2022) [44] evaluated the use of WTTF fibers as an additive to improve the rheological properties of asphalt cement and the performance of mixtures, obtaining significant improvements, especially at high service temperatures. However, the addition of WTTF as a fiber to the asphalt mixture or to the asphalt binder is not a good option for industrial application due to the formation of clusters, which prevent a homogeneous distribution in the mixture matrix. In this context, Valdés et al. (2022) [23] developed a new granular additive based on WTTF fibers for HMA mixtures, obtaining significant improvements in the stiffness modulus, resistance to permanent deformation, and moisture damage. Likewise, Valdés et al. (2023) [45] evaluated a new format of this WTTF-based additive as a stabilizer for SMA mixtures, obtaining design and performance properties comparable to the reference SMA mixture with cellulose fibers. The granular format of the additive is an innovative characteristic that aims to facilitate technological transfer to the asphalt paving industry. The granular format makes it possible to facilitate the dosing processes, addition to the mixture in the production process, and homogeneous distribution of the additive in the mixture.

These previous results support the use of WTTF-based additives to improve the performance properties of HMA asphalt mixtures or to be a good substitute for the commercial cellulose stabilizing additive used in SMA mixtures. Due to these promising results, this research assesses the effect of this new additive on the critical property of fatigue, which has not been studied. Fatigue cracking is a critical phenomenon that influences the structural strength and service life of pavements, so it is important to evaluate the effect of this WTTF-based additive on this property. For this purpose, two reference asphalt mixtures, HMA/R and SMA/R, were designed. Then, the effect of the addition of both WTTF-based additive formats on the designing properties and fatigue cracking resistance under controlled cyclic loading conditions was evaluated. In addition, a fatigue life analysis was performed using an empirical–mechanistic design methodology. This made it possible to evaluate the durability of a pavement structure with varying thicknesses of asphalt mix layers.

## 2. Materials and Methods

### 2.1. WTTF-Based Additive

**Figure 1:**
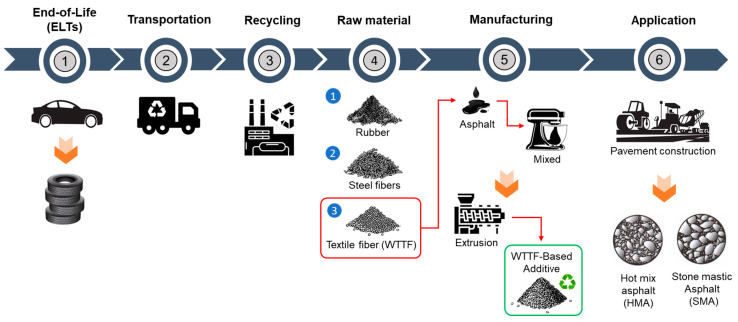
WTTF-based additive manufacturing process.

In previous studies, two formats of a new additive developed based on waste tire textile fiber (WTTF) were evaluated for use in the hot-mix asphalt (HMA) [23] and stone mastic asphalt (SMA) [45]. The raw material of the additive is composed of the mixture of WTTF, cationic rapid-setting asphalt emulsion, and water in a weight ratio of 1:1:1. During the drying process of the raw material, the water evaporates as part of the emulsion breaking process. Subsequently, the resulting mass is extruded and cut to shape the additive, thus transforming the raw material into pellets. To avoid cohesion between the pellets, rubber powder is added in a 1:20 weight ratio (rubber powder: pellets). The final composition of the developed WTTF-based additive was 58% (by weight) WTTF, 37% (by weight) asphalt cement, and 5% (by weight) rubber powder (European Patent N°4008753). Two ways of manufacturing the additive to be incorporated into the HMA and SMA mixtures were evaluated: (1) an additive obtained via a manual process at a laboratory scale; and (2) an additive obtained by a mechanized process at an industrial scale. The manual process of manufacturing the additive consists of kneading the fiber coated with asphalt binder until the desired shape and density of the cylinder are achieved. The cylinders are then subjected to guillotine cutting. Finally, the additive is packaged after conditioning with fine rubber powder. In the mechanized process, the asphalt binder-coated fiber is extruded at a pressure of 3000 PSI (with one device for the additive intended to be used in the HMA mixture and another device for the additive intended to be used in the SMA mixture). Then, the extruded material is subjected to a cutting and conditioning process with fine rubber powder prior to packaging. The manufacturing process of the WTTF-based additive is detailed in Figure 1. In addition, Table 1 and Table 2 show the physical characteristics of the WTTF-based additive used in HMA and SMA mixes, respectively.

### 2.2. Commercial Cellulose Additive

A commercial cellulose additive was used as a stabilizing agent in SMA-type asphalt mixtures. This additive is composed of 34% 50/70 asphalt and 66% natural cellulose fibers [46]. The main characteristics of the commercial cellulose additive are shown in Table 3.

### 2.3. Asphalt Binder

In this study, two types of asphalt binders were used. The first is a conventional asphalt binder of type CA-24, which was used in the HMA asphalt mixtures. The second is a polymer-modified asphalt binder of type CA-60/80, which was used in the SMA asphalt mixtures. Both types of asphalt binders were classified according to the Chilean Standard [47]. The properties of these asphalt binders are shown in Table 4 and Table 5.

### 2.4. Aggregates

The aggregates used in this study are of fluvial origin and meet the requirements established by the Chilean Standard for wearing course [48]. The composition of the aggregates includes dolomite, basalt, dacite, andesite, rhyolite, sandstone, quartz, and quartzite particles. In the case of SMA-type asphalt mixtures, 8% mineral filler (lime) was used in relation to the weight of the aggregates. The properties of the aggregates used are shown in Table 6.

**Figure 2:**
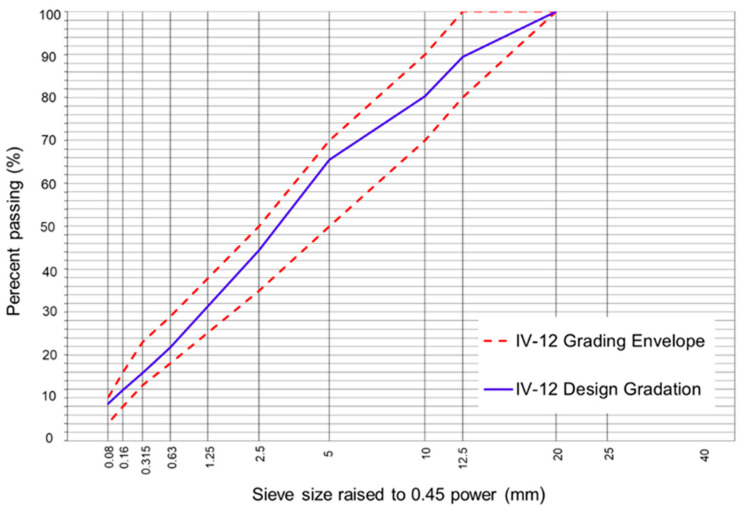
Design gradation of the IV-12 asphalt mixture.

**Figure 3:**
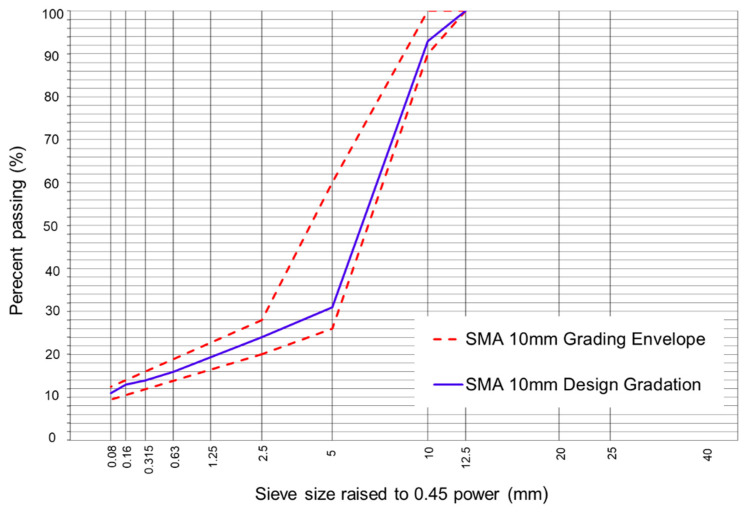
Design gradation of the SMA10 asphalt mixture.

The aggregate particle sizes were specified according to the Chilean Standard. For HMA asphalt mixtures, the aggregate fractions were adjusted according to a dense-grain-size band type IV-12 [48]. In the case of SMA asphalt mixtures, the aggregate fractions, including the mineral filler, were adjusted according to a particle size band with a maximum nominal size of 10 mm (SMA10) [49]. The gradation of the aggregates used in the HMA and SMA asphalt mixtures can be seen in Figure 2 and Figure 3, respectively.

## 3. Experimental Design

**Figure 4:**
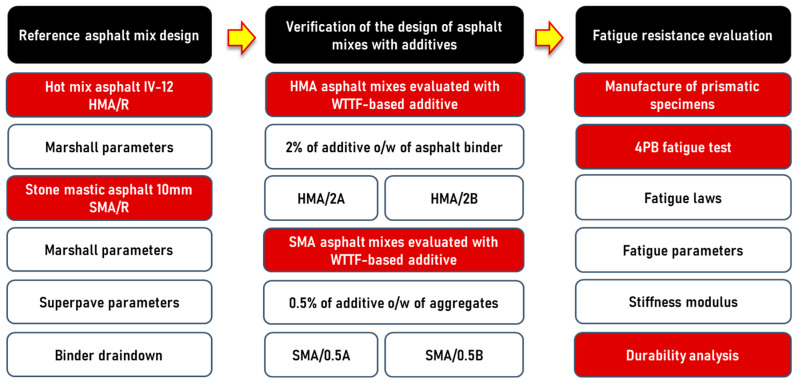
Experimental stages used in this study.

The experimental design performed in this study is detailed in Figure 4. First, the design of the HMA/R and SMA/R reference asphalt mixtures was performed based on the design criteria established by Chilean Standards. Second, the effect of the addition of both additive formats based on WTTF on the design properties, produced manually and mechanized, was evaluated. For the HMA mixture, the effect of an optimum addition percentage of 2% WTTF-based additive on the weight of asphalt cement was evaluated [23]. For the SMA mixture, the effect of the addition of 0.5% WTTF-based additive on the aggregate weight (equivalent to 100% replacement of the commercial cellulose additive used in the reference mix) was evaluated [45]. Third, the performance of the studied mixtures was evaluated in terms of fatigue resistance by means of the four-point bending test (4PB). In addition, an empirical–mechanistic analysis was performed to evaluate the durability of a pavement-type structure with variable thickness of asphalt layers.

### 3.1. Asphalt Mix Design

The design of the reference HMA and SMA asphalt mixtures, designated as HMA/R and SMA/R, respectively, was performed based on the specifications of the Chilean Standard [48]. The optimum asphalt contents determined for the HMA/R and SMA/R mixtures were 5.3% and 6.8%, respectively, over the weight of aggregates. In the case of the SMA/R mixture, 0.5% of commercial cellulose additive was used.

In this study, different WTTF-based additive contents were selected to be evaluated in the performance properties of HMA and SMA mixtures while maintaining the optimum asphalt content determined for each reference mixture. For the HMA asphalt mixtures, an optimum content of 2% WTTF-based additive on the weight of asphalt cement was used for both the manually and mechanically manufactured additive, hereafter referred to as HMA/2A and HMA/2B, respectively. In the case of the SMA asphalt mixtures, the total replacement of the commercial cellulose additive content used in the mix was evaluated by using the WTTF-based additive at a proportion of 0.5% by the weight of the aggregates. This evaluation was performed for both the manually and mechanically manufactured additives, hereafter referred to as SMA/0.5A and SMA/0.5B, respectively. Both optimum contents of WTTF-based additive used in the HMA and SMA mixtures were obtained from two previous studies [23,45]. The design parameters of the evaluated mixtures are shown in Table 7. The results of the parameters obtained from the Marshall method: stability, flow, air voids, and voids in mineral aggregate (VMA) for the HMA mixtures comply with the design values established in the Chilean standards. Likewise, it is observed that in the SMA mixtures, the parameters of air voids, VMA, air voids in the coarse aggregate of the aggregate mix (${VCA}_{MIX}$), air voids in the compacted coarse aggregate (${VCA}_{DRC}$), and binder drainage adhere to the design criteria established in the Chilean standards.

### 3.2. Preparation of Asphalt Mix Samples

**Figure 5:**
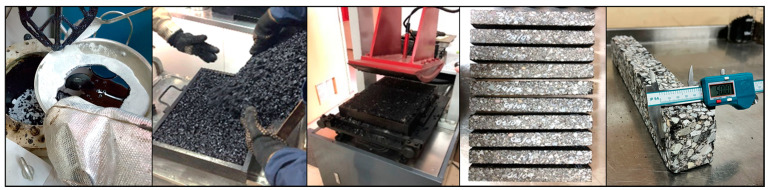
Preparation of prismatic test specimens.

In this study, prismatic specimens were prepared according to the European standard EN 12697-33 [51]. The specimen dimensions were as follows: *b* = 50 mm; *h* = 50 mm; *L* = 400 mm (average distance between the outer clamps). Initially, the materials were mixed in a mechanical mixer at the mixing temperature defined for HMA and SMA mixtures (154 °C and 177 °C, respectively), ensuring a homogeneous distribution between the WTTF, the stone aggregates, and the asphalt binder. The evaluated mixtures were placed in a metal mold conditioned at the compaction temperature defined for the HMA and SMA mixtures (145 °C and 165 °C, respectively), where asphalt slabs of asphalt mixture were obtained at the design density by means of an asphalt slab roller compactor. In the HMA and SMA mixtures, an average of 33 and 44 compaction cycles were applied, respectively. Prismatic samples were prepared, verifying their volumetric properties, to be tested 2 weeks after the cutting date. Figure 5 shows the process developed for the manufacture of the test specimens.

### 3.3. Testing Methods

**Figure 6:**
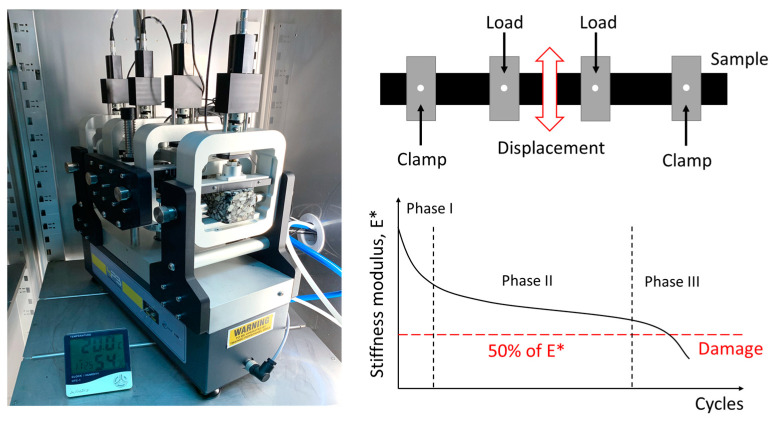
4PB fatigue test described in EN 12697-24, Annex D [52].

To evaluate the effect of the WTTF-based additive on the fatigue resistance of HMA and SMA asphalt mixtures, the four-point bending beam (4PB) test was performed according to the European standard EN 12697-24, Annex D [52]. This test consists of subjecting a section of a prismatic beam to constant stress until failure occurs (Figure 6). All specimens were evaluated in an environmental chamber at a temperature of 20 °C, with a preconditioning of 4 h. To determine the fatigue laws, three strain levels were evaluated at a constant frequency of 10 Hz, considering at least a total of six specimens per level. In the case of the HMA mixtures, 150, 190, and 300 microstrain levels were evaluated, while for the SMA mixtures, 400, 500, and 700 microstrain levels were evaluated. Additionally, the value of ε (10^6^) used to characterize the fatigue resistance of asphalt mixtures was calculated [53,54]. The coefficient of determination (R^2^) was used as an indicator of the quality of fit. The fatigue curve was determined using the least squares regression relationship, Equation (1).

(1) $$\varepsilon =a\;*\;N^{-b}$$

where *ε* is the tensile strain; *N* is the number of cycles to failure, and *a* and *b* are coefficients of the fatigue laws. The progressive process of fatigue damage is addressed in three damage phases. In phase I, also known as the adaptation phase, the modulus decreases rapidly with increasing load cycles, accounting for approximately 10% of the fatigue life of the specimen [34,55,56]. In phase II, referred to as the fatigue phase, the modulus decreases linearly with increasing load cycles. At the end of this phase, the specimen reaches approximately 90% of its fatigue life [34,55,56]. In phase III, or the rupture phase, the modulus decreases abruptly with the loading cycles, which eventually leads to total failure of the specimen [34,55,56]. During the final phase, the stiffness of the specimen may remain nearly constant for long periods of time before reaching its failure point. For this reason, the fatigue failure criterion is defined in terms of the percentage reduction of 50% of the initial stiffness measured in cycle 100 [52,56]. During the test, fatigue parameters such as phase angle (δ) and dissipated energy (J/m^3^) were recorded. The phase angle (δ) allows for relating the lag generated between the applied load and the strain generated by that load. The dissipated energy is an indicator for measuring the toughness of the mixture.

The stiffness modulus (*S_M_*) was determined by means of the indirect tensile test (ITS) based on the European standard EN 12697-26 [57]. The test consists of applying load pulses on one of the diametral planes of a cylindrical specimen, recording the applied load variation and the horizontal diametral strain over time, together with the determination of the loading surface factor. A total of three cylindrical specimens were manufactured for each type of evaluated mixture, which were kept at a controlled temperature of 20 °C for a period of 24 h before testing. The calculation of the stiffness modulus (*S_M_*) was determined by means of Equation (2).

(2) $$S_{M}=\frac{F\cdot (v+0.27)}{\left(z\cdot h\right)}$$

where *S_M_* is the stiffness modulus measured in (MPa); *F* is the maximum vertical load applied in (N); *v* is Poisson’s ratio; *z* is the horizontal displacement in (mm), and *h* is the average thickness of the specimen in (mm).

**Figure 7:**
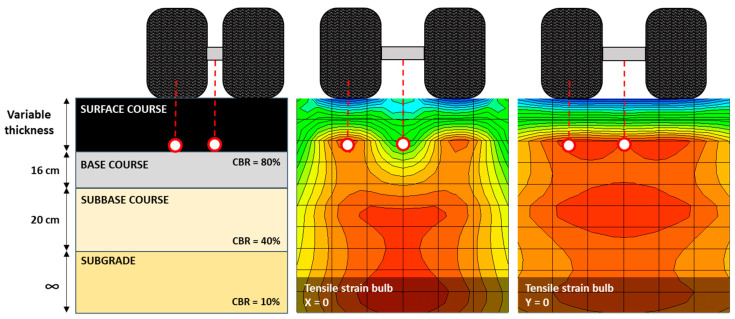
Structure and fatigue analysis points.

An empirical–mechanistic analysis was performed to model the behavior of pavement structures. Tensile strains were measured at critical points for different pavement layer thicknesses. The evaluated structure is composed of an asphalt mixture layer of variable thickness (5 to 30 cm), a 16 cm granular base, and a 20 cm granular subbase supported on the subgrade. Moduli of 327 MPa, 167 MPa, and 77 MPa were established for the granular base, granular subbase, and subgrade layers, respectively. The stress and strain response of the evaluated pavement structures was determined using MePADS software version number 1.1. To determine the characteristics of the structure, the stiffness modulus, layer thickness, and Poisson’s ratio (0.35 for both mixtures) were considered as input variables. Two loads were evaluated for a single axle with double wheels: 8.16 Ton (equivalent to 80 kN), which is traditionally used in pavement design; and 11 Ton (equivalent to 110 kN), which is the maximum weight allowed in Chile for the type of axle evaluated. The critical points were evaluated, both under wheel and between wheels, as shown in Figure 7.

### 3.4. Statistical Analysis

An analysis of variance (ANOVA) was performed to evaluate the statistical significance of the durability results of the asphalt mixtures obtained from the empirical mechanistic analysis. Three variables were considered in the analysis: the thickness of the wearing course; the additive format; and the axle loads. An ANOVA was performed at a 95% confidence level. Two statistical parameters, the *p*-value and F-value, were used to evaluate the acceptance or rejection of the null hypothesis $(H_{0}$) of equality of variance. The *p*-value was used to determine the significance of each variable in relation to the result of the parameter evaluated, considering a *p*-value of less than 0.05 as statistically significant. In addition, the F-value was used to evaluate the degree of significance, where a higher F-value indicated a greater influence of the variable on the result.

## 4. Analysis of the Results

### 4.1. Results of HMA Mixtures

**Figure 8:**
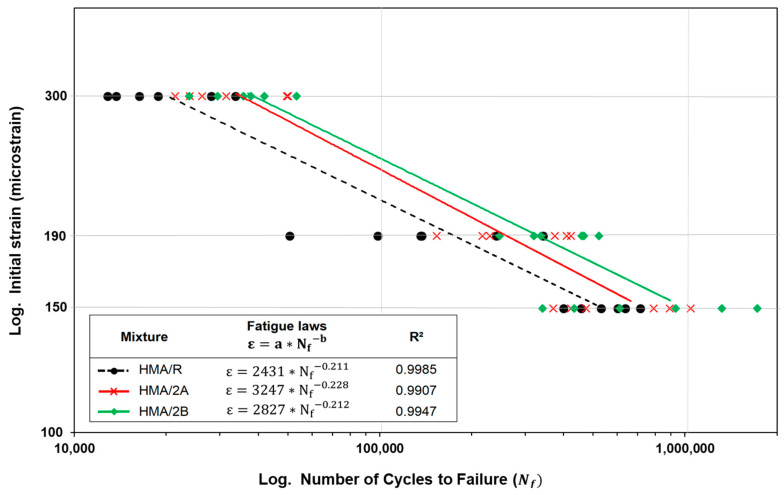
Fatigue laws of HMA mixtures obtained via the 4-point fatigue test.

**Figure 9:**
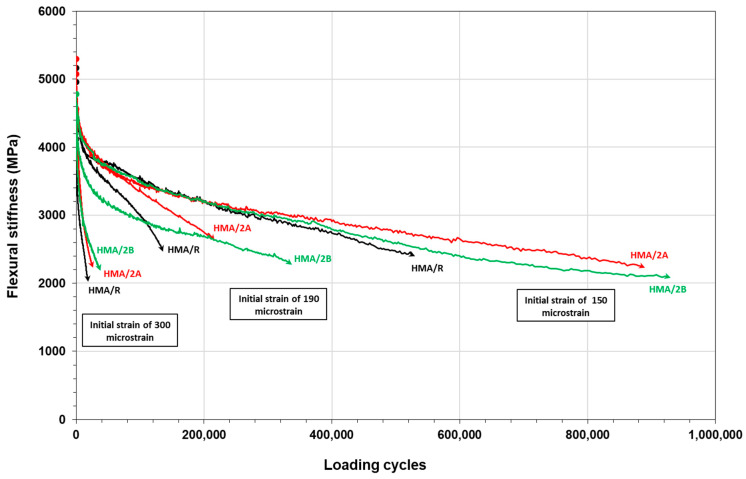
Flexural stiffness curve in HMA mixtures at different strain levels.

Figure 8 shows the fatigue laws obtained for the HMA mixtures. According to the results obtained, it is observed that the addition of 2% WTTF-based additive content, both manual (HMA/2A) and mechanized (HMA/2B), improves the fatigue performance in relation to the reference mixture (HMA/R). The fatigue laws show that the HMA/2A and HMA/2B mixtures increased their resistance to the application of load cycles for the same strain levels applied to the tested samples with respect to the HMA/R mixture by 54.5% and 91.4% on average, respectively. These results are consistent with the studies of Tapkin (2008) [58] and Taherkhani (2017) [59], who evaluated the incorporation of synthetic fibers in asphalt mixtures, determining an increase in fatigue resistance of around 27–33%, with respect to the reference mixture. This is related to the effect generated by the WTTF in the aggregate-binder matrix, forming a kind of three-dimensional network that improves the integrity, stress dispersion, and delay in the extension of microcracks, as mentioned in the study by Li et al. (2020) [60]. The effect of the incorporation of the WTTF additive was lower at low-strain levels (150 microstrains), with a greater effect on fatigue resistance at intermediate- and high-strain levels (190 and 300 microstrains). In the case of the HMA/2A mixture, the average fatigue resistance of the specimens tested was increased by 19.5%, 79.7%, and 64.7% for the strain levels of 150, 190, and 300 microstrains, respectively. While in the HMA/2B mixture, the fatigue resistance was increased by 60.4%, 133.9%, and 79.9%, for the strain levels of 150, 190, and 300 microstrains, respectively. According to the coefficient of determination (R^2^), the indicated value of R² was of the order of 0.99, indicating a good correlation between initial strain and the loading cycles for the HMA mixtures studied. This may suggest that HMA mixtures with WTTF-based additives may increase their fatigue damage performance (relative to the mixture without the use of the admixture). Additionally, Figure 9 shows the evolution of the flexural stiffness of the HMA mixtures evaluated at different strain levels. The results show that the initial flexural stiffness gradually decreases as the number of loading cycles increases until failure occurs. It is observed that the higher the level of strain, the earlier the failure occurs, regardless of the type of mixture evaluated. When comparing the reference mixture with the mixtures with the use of the WTTF-based additive, it is observed that the HMA/2A and HMA/2B mixtures present a greater capacity to withstand load cycles since they registered a greater number of cycles to reach the failure limit point related to the 50% reduction in the initial modulus, for the different strain levels evaluated.

On the other hand, the value of ε (10^6^) was determined, which indicated the strain level at which the asphalt mixture reached fatigue-induced failure after one million load cycles. The results show that the use of the WTTF additive gives the mixture an improvement in flexural capacity and energy absorption against loads, which is reflected in an increase in the strain value at one million load cycles. It was observed that the HMA/2A and HMA/2B mixtures presented ε (10^6^) values in the order of 139.1 and 151.1 μm/m, respectively, which were higher than the value of 131.7 μm/m determined for the HMA/R mixture. A similar effect to that observed by Saliani et al. (2021) [53], where higher values of Ɛ (10^6^) were obtained by adding a synthetic fiber to the asphalt mixture compared to the reference mixture. This effect is related to that determined by Guo et al. (2020) [61], who concluded that the use of mineral or synthetic fibers in asphalt mixtures could delay the evolution of cracking and prolong the phase of elastic behavior, which would explain the extension of the strain level for the same number of cycles.

The results of the fatigue parameters initial strain, initial phase angle, final phase angle, and dissipated energy obtained for the HMA mixtures in the 4PB test are shown in Table 8. In relation to the phase angle (δ), an increase in the parameter δ can be observed at higher strain levels in all the mixtures evaluated. This behavior is explained by the fact that a higher applied stress is required (to achieve the defined strain level), which enhances the viscous component in the rheological behavior of the asphalt binder, similar to the behavior occurring at low loading rates or high temperatures. Consequently, it is observed that as the applied strain decreases, the value of parameter δ also decreases. On the other hand, when considering all the strain levels evaluated, the HMA/2A and HMA/2B mixtures recorded an average increase in the differences between the initial phase angle and the final phase angle with respect to the HMA/R mixture of 12.1% and 27.2%, respectively. This behavior indicates that there is an increase in the flexibility range of the evaluated mixtures that used the WTTF-based additive. This effect may be due to the reinforcement generated in the asphalt mixture matrix as a result of the fiber-mastic bonding properties, which would contribute to improving the ductility of the mixture and could explain the increase in the number of cycles to failure. The evaluation of the dissipated energy indicates that the HMA/2A and HMA/2B mixtures require a greater amount of work and load cycles to achieve material failure relative to the HMA/R reference mixture. This suggests that the WTTF additive may provide greater tenacity to the mixture, resulting in an increased ability to withstand repetitive stresses. This effect is in agreement with that observed in the studies of Lou et al. (2021) and Kim et al. (2018), who analyzed polypropylene, polyester, nylon, and carbon fibers, which improved the flexibility and tenacity properties of the mixture [62,63].

**Figure 10:**
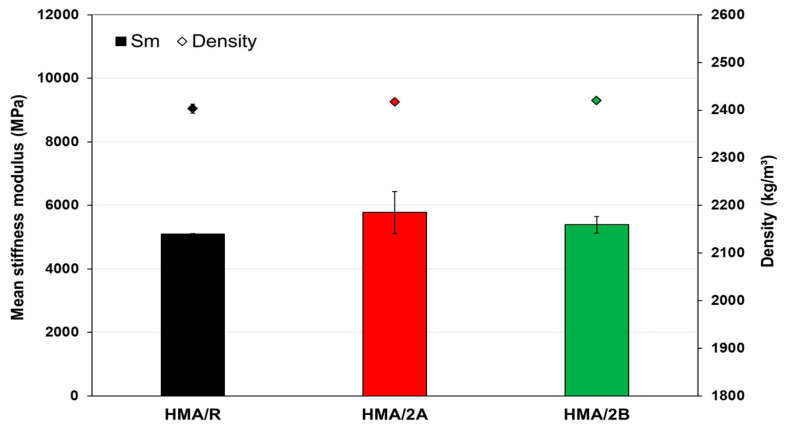
Stiffness modulus at 20 °C for HMA mixtures.

The results obtained for the stiffness modulus of the HMA mixtures via the ITS test are shown in Figure 10. The results indicate that the HMA/2A and HMA/2B mixtures present an average stiffness modulus value higher than that of HMA/R by 14.9% and 7.2%, respectively. These results indicate that the WTTF additive would increase the internal cohesive forces of the asphalt mixture. This observed effect coincides with previous studies that have researched the addition of different types of synthetic fibers in asphalt mixtures [64,65,66]. The aforementioned is relevant for asphalt mixtures since a higher stiffness can translate into a higher structural capacity of the pavement [67].

### 4.2. Durability Results of the Pavement Structure of HMA Mixtures

**Figure 11:**
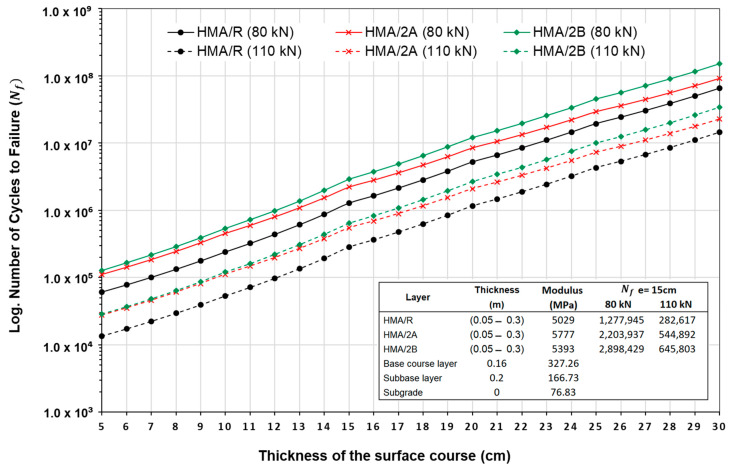
Durability of the pavement structure of HMA mixtures for different thicknesses and traffic loads.

Figure 11 shows the results of pavement structure durability of the HMA mixtures evaluated for different asphalt mixture layer thicknesses and traffic loads. The results show that the evaluated mixtures obtain higher tensile strains under the 110 kN axle load compared to the 80 kN axle load. Therefore, the durability of the pavement structure for the different thicknesses evaluated decreases to a greater extent for the 110 kN axle. Comparing the durability of the mixtures at an asphalt wearing course thickness of 15 cm, it was observed that for an axle load of 80 kN, the HMA/2A and HMA/2B mixtures increased the durability relative to the reference HMA/R mixture by 72.5% and 126.8%, respectively. Likewise, at higher loads of 110 kN, the HMA/2A and HMA/2B mixtures increased the durability relative to the HMA/R reference mixture by 92.8% and 128.5%, respectively. When specifically analyzing the durability for 1,000,000 load cycles, it is observed that by using the WTTF-based additive, it is possible to reduce the wearing course of the mixture by 2 to 3 cm without compromising the fatigue performance of the mixture compared to the HMA/R mixture. This increase in durability is related to the results obtained in the stiffness modulus and fatigue laws. On the one hand, the increase in the stiffness modulus in the HMA mixtures with WTTF additive suggests that they could obtain lower tensile strains under the axle loads evaluated. On the other hand, fatigue laws show that these mixtures are able to withstand a higher number of load cycles before failure.

### 4.3. Results of SMA Mixtures

**Figure 12:**
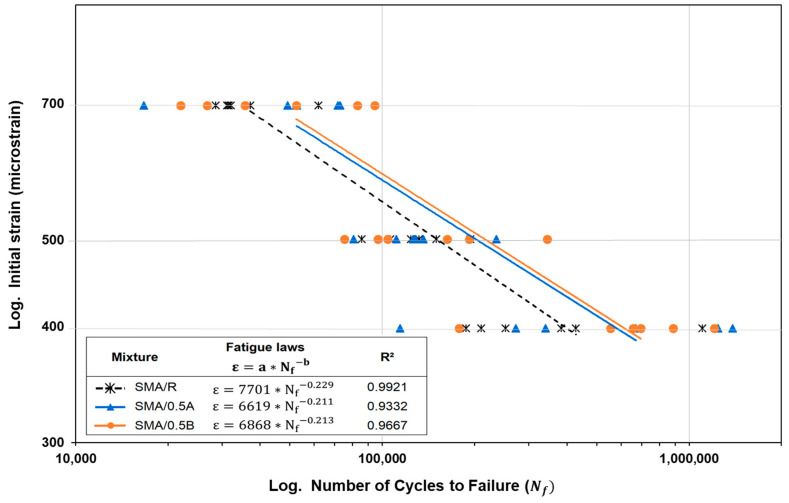
Fatigue laws of SMA mixtures obtained via the 4-point fatigue test.

Figure 12 presents the results obtained for the fatigue laws of the SMA mixtures. From the fatigue laws, an average increase in load cycles to failure is observed for the evaluated strain levels of the SMA/0.5A and SMA/0.5B mixtures in relation to the SMA/R reference mixture. These increases are 34.1% and 42.5%, respectively. These results are in agreement with those obtained in the study of Mahrez and Karim (2010) [68], in which glass fiber was incorporated into the SMA mixtures, observing an increase in fatigue life in the range from 10% to 79% compared to the control mixture. In relation to the strain levels applied in the 4PB fatigue tests, it was observed that for the lowest strain level evaluated (400 microstrains) the SMA/0.5A and SMA/0.5B mixtures registered a greater difference in the number of application cycles until failure was reached than for the highest strain levels (500 and 700 microstrains), compared to the SMA/R mixture. For the SMA/0.5A mixture, an increase in load cycles to fatigue failure of 57.1%, 3.4%, and 41.8% was recorded for the strain levels of 400, 500, and 700 microstrains, respectively. Similarly, the SMA/0.5B mixtures also showed an increase of 62.9%, 23.2%, and 41.2%, respectively, for the same strain levels. According to the coefficient of determination (R^2^), the indicated value of R^2^ was in the order of 0.93–0.99, indicating a strong correlation between initial strain and fatigue life for the SMA mixtures studied. These results obtained show the potential of the WTFF-based additive to be used in SMA mixtures as a replacement for the commercial cellulose additive since the increase in resistance to the application of load cycles for each level of strain evaluated indicates a greater capacity to resist fatigue damage compared to the commercial cellulose additive used in the SMA/R mixture.

**Figure 13:**
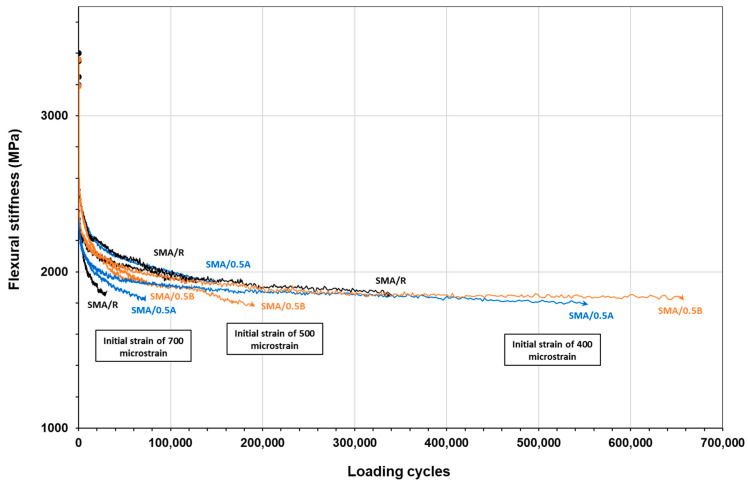
Flexural stiffness curve in SMA mixtures at different strain levels.

Figure 13 shows the evolution of the flexural modulus of the SMA mixtures as a function of load cycles for each type of mixture evaluated at different strain levels. The results indicate that the SMA/0.5A and SMA/0.5B mixtures showed a higher capacity to resist the load cycles compared to the SMA/R mixture. These mixtures took longer to reach the failure point, which is defined as a 50% reduction in the initial modulus, especially at low strain levels of 400 microstrains. These results were consistent with the value of ε (10^6^), where it was obtained that the SMA/0.5A and SMA/0.5B mixtures presented higher values than the SMA/R mixture, which indicates a greater capacity to resist strains under the same level of load application corresponding to one million cycles. Specifically, ε (10^6^) values in the order of 325.5, 358.8, and 362.1 μm/m were recorded for the SMA/R, SMA/0.5A, and SMA/0.5B mixtures, respectively.

**Figure 14:**
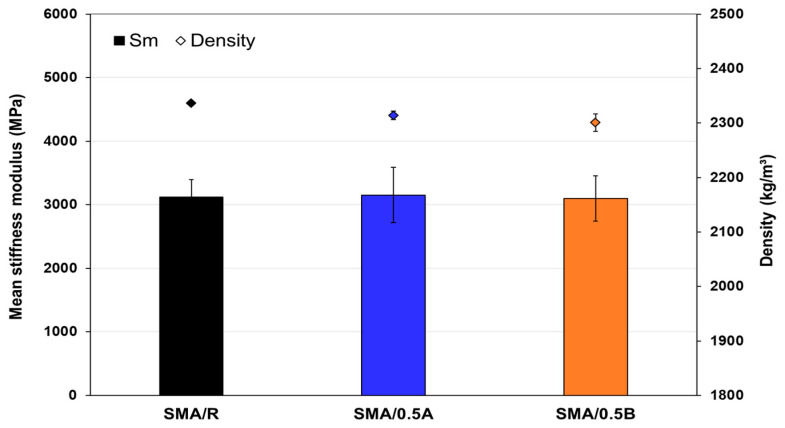
Stiffness modulus at 20 °C for SMA mixtures.

The results of the fatigue parameters obtained for the SMA mixtures by the 4PB test are shown in Table 9. In relation to the phase angle (δ), it was observed that the SMA/0.5A and SMA/0.5B mixtures presented an average increase of 49.7% and 38.6%, respectively, in the range of the initial and final phase angle parameter, compared to the SMA/R mixture. These results indicate that the use of the WTTF-based additive produces an increase in the flexibility range of the SMA mixtures during fatigue damage. Furthermore, due to the analysis of the dissipated energy parameter, it is shown that the SMA/0.5A and SMA/0.5B mixtures require a greater amount of work to reach mixture failure in relation to the SMA/R mixture. This indicates that the use of WTTF-based additive in SMA mixtures not only serves the function of stabilizing the asphalt binder in the mixture matrix, but also provides a greater capacity to resist repetitive stresses. The stiffness modulus results obtained in the ITS test, shown in Figure 14, indicate that the SMA/0.5A and SMA/0.5B mixtures performed similarly to the SMA/R mixture. This effect is in agreement with what has been observed in other studies, which support the idea that the use of fibers of mineral and synthetic origin in SMA mixtures reinforces the aggregate-binder matrix [41,69]. This suggests that the WTTF-based additive, with polymeric fibers of synthetic origin [8], could generate a comparable effect on the stiffness modulus property to the commercial cellulose admixture used in the reference mixture.

### 4.4. Durability Results of the Pavement Structure of SMA Mixtures

**Figure 15:**
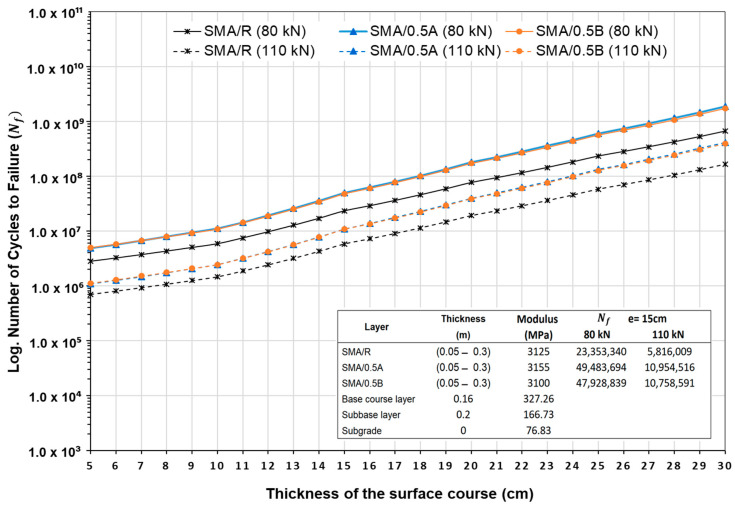
Durability of the pavement structure of SMA mixtures for different thicknesses and traffic loads.

Figure 15 presents the durability results of the pavement structure for the SMA mixtures evaluated for different asphalt mixture layer thicknesses and for two-axle loads of 80 kN and 110 kN. When comparing the durability of the mixtures at an average thickness of 15 cm, it was observed that under a load of 80 kN, the SMA/0.5A and SMA/0.5B mixtures showed a better performance in relation to the SMA/R mixture, with significant increases of 111.9% and 105.2%, respectively. Similarly, when increasing the loads to higher levels of 110 kN, the SMA/0.5A and SMA/0.5B mixtures also show an increase in durability, with increases of 88.4% and 85.0%, respectively. However, when analyzing the performance in terms of durability in relation to the additive format used, it is observed that both SMA/0.5A and SMA/0.5B mixtures reached failure in similar cycles. On the other hand, when evaluating the durability specifically for 10,000,000 load cycles, it is observed that the use of WTTF-based additive in SMA mixtures would allow for a reduction of 2 to 3 cm in the thickness of the wearing course, obtaining the same fatigue performance as the SMA/R reference mixture. These results coincide with those obtained in HMA mixtures evaluated with a WTTF-based additive. However, it is important to note that the SMA mixtures showed better performance in terms of durability compared to the HMA mixtures. For the same thickness, SMA mixtures were able to withstand a higher number of cycles before reaching failure, indicating a longer fatigue life. This behavior is mainly attributed to the inherent characteristics of SMA mixtures [27,28,41,70].

Table 10 presents the statistical analysis of the results obtained from the empirical mechanistic analysis related to the fatigue durability of asphalt mixtures. According to the analysis of variance (ANOVA), the null hypothesis $\left(H_{0}\right)$ of equality of variances is rejected, with a *p*-value of less than 0.05. This indicates that the WTTF-based additive has a significant effect on the fatigue durability of HMA and SMA mixtures. These results statistically demonstrate that the WTTF-based additive, in both formats, improves the performance of asphalt mixtures in terms of resistance to repetitive load cycles, which indicates an increase in the fatigue durability of asphalt mixtures. In addition, a high statistical significance is observed in relation to the type of axle load, supported by higher F-values. This indicates that the type of load applied has a greater influence on the durability of asphalt mixtures.

## 5. Conclusions

The manually and mechanically manufactured WTTF-based additive, in its two formats, can be used in both HMA and SMA mixtures, improving their properties in terms of resistance to fatigue damage and increasing the fatigue durability of pavements.

1. Fatigue laws showed that the use of the WTTF-based additive allowed for improving the resistance of HMA and SMA mixtures to fatigue damage. These mixtures indicated a higher capacity to withstand load cycles before reaching failure, with ε (10^6^) values higher than those of the reference mixtures;
2. The use of the WTTF-based additive in HMA and SMA mixtures indicated an improvement in the viscoelastic property of the mixture. As a result, these mixtures showed increased flexibility with a slower reduction in stiffness as load cycles were applied, which indicated a greater ability to dissipate energy during the cracking process;
3. Regarding the stiffness modulus at 20 °C, the incorporation of the WTTF-based additive in the HMA mixture generates an increase in its stiffness modulus value compared to the reference mixture, which contributes to the pavement structure. In the case of the SMA mixture, the WTFF-based additive showed stiffness modulus values similar to those of the reference mixture;
4. The durability evaluation showed that all mixtures with WTFF-based additive presented increases in durability compared to the reference mixtures, both at different pavement thicknesses and for both types of axle loads evaluated;
5. ANOVA showed a significant effect of WTTF-based additive on the performance of the HMA and SMA asphalt mixtures versus the fatigue performance of mixtures in a pavement structure;
6. This research demonstrates the potential of the WTTF-based additive to optimize the durability of asphalt mixtures. This additive, developed from a massive by-product of the tire-recycling industry, not only promotes sustainability but also reduces the need for virgin raw material in SMA mixtures.

The results obtained in this experimental study have allowed us to demonstrate the positive and statistically significant effect that the WTTF-based additive has on the fatigue performance of HMA and SMA asphalt mixtures. The format of the developed additive would allow for an easy industrial application in asphalt plants and contribute to improving the circular economy of the tire industry. The main contribution of this study is that a product has been developed to improve the performance of asphalt pavements with an emphasis on sustainability and material reuse in a format that facilitates its industrial application.

## 6. Recommendations

It is recommended to conduct a full-scale test section, which would validate the contribution of the additive in improving the performance properties of the evaluated mixtures. Additionally, it would be interesting to evaluate the social and environmental contribution of the use of WTTF-based additives in asphalt mixtures.

## Figures and Tables

**Table 1:** Characterization of the WTTF-based granular additives evaluated in HMA mixtures.

Manual Manufacturing		Mechanical Manufacturing	
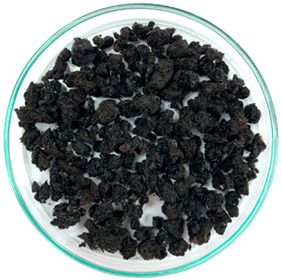		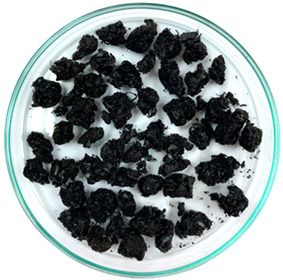	
**Characteristic**	**Description**	**Characteristic**	**Description**
Diameter range (mm)	3.1–7.2	Diameter range (mm)	4.7–9.9
Bulk density (g/cm³)	0.15	Bulk density (g/cm³)	0.16
Real density (g/cm³)	1.15	Real density (g/cm³)	1.14

**Table 2:** Characterization of the WTTF-based granular additives evaluated in SMA mixtures.

Manual Manufacturing		Mechanical Manufacturing	
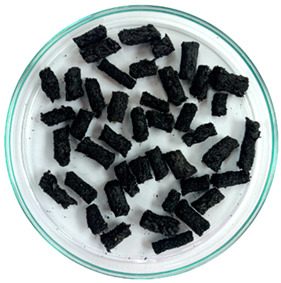		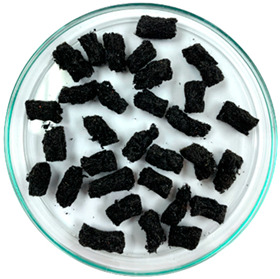	
**Characteristic**	**Description**	**Characteristic**	**Description**
Diameter range (mm)	3.6–5.8	Diameter range (mm)	4.9–6.8
Length range (mm)	4.8–12.1	Length range (mm)	6.3–14.2
Bulk density (g/cm³)	0.24	Bulk density (g/cm³)	0.19
Real density (g/cm³)	1.18	Real density (g/cm³)	1.14

**Table 3:** Characterization of the commercial cellulose additive.

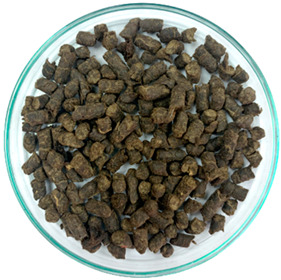	
**Characteristic**	**Description**
Diameter range (mm)	4.0–4.8
Length range (mm)	4.3–12.4
Apparent density (gr/cm³)	0.38
Real density (gr/cm³)	1.49

**Table 4:** Properties of the asphalt binder used in HMA mixtures.

Tests	CA-24	Specs. [47]
Absolute viscosity at 60 °C, 300 mm Hg (P)	3072	Min. 2400
Penetration at 25 °C, 100 g. 5 s. (0.1 mm)	58	Min. 40
Ductility at 25 °C (cm)	>150	Min. 100
Spot test hep/xyl. (%xylene)	<30	Max. 30
Cleveland open cup flash point (°C)	322	Min. 232
Softening point (Ring and Ball) (°C)	51.4	To be reported
Trichloroethylene solubility (%)	99.9	Min. 99
Penetration index	−0.1	−2.0 a + 1.0
RTFOT (Rolling Thin-Film Oven Test)		
Mass loss, (%)	0.42	Max. 0.8
Absolute viscosity at 60 °C, 300 mm Hg (P)	10,933	To be reported
Ductility at 25 °C, 5 cm/min (cm)	>150	Min. 100
Durability index	3.6	Max. 4.0
Mixing temperature at 2 Poise (°C)	154 ± 5	
Compaction temperature at 3 Poise (°C)	145 ± 5	

**Table 5:** Properties of the asphalt binder used in SMA mixtures.

Tests	CA 60/80	Specs. [47]
Penetration at 25 °C, 100 g. 5 s. (0.1 mm)	62	60–80
Softening point (Ring and Ball) (°C)	72.4	Min. 60
Ductility at 25 °C, 5 cm/min, (cm)	112	Min. 80
Linear elastic recovery at 13 °C, 20 cm, 1 h (%)	85	Min. 50
Elastic recovery by torsion at 25 °C (%)	72	Min. 60
Penetration index	3.8	Min. +2.0
FRAASS breaking point (°C)	−15	Max. −15
Flash point (°C)	>300	Min. 235
Storage stability	<4	Max. 5.0
Performance grade PG	64V(22)-28	To be reported
Mixing temperature at 2 Poise (°C)	177 ± 5	
Compaction temperature at 3 Poise (°C)	165 ± 5	

**Table 6:** Physical properties of the aggregates used.

Tests	HMA	SMA	Specs. [48]
Coarse aggregate			
Los Angeles abrasion loss (%)	20	14	Max. 25
Sodium sulfate soundness (%)	2.4	0.3	Max 12
Crushed aggregates (%)	96	96	Min. 90
Flaky aggregates (%)	1	0.5	Max. 10
Static adhesion method	>95	>95	Min. 95
Dynamic adhesion method	>95	>95	Min. 95
Specific gravity (kg/m^3^)	2685	–	–
Absorption (%)	1.54	–	–
Fine aggregate			
Plasticity index	Non-plastic	Non-plastic	Non-plastic
Riedel-Weber adhesion	3–10	4–9	Min. 0–5
Sodium sulfate soundness (%)	1.4	1.0	Max. 15
Specific gravity (kg/m^3^)	2650	–	–
Absorption (%)	1.1	–	<3
Combined aggregate			
Soluble salts (%)	0	0	Max. 2
Sand equivalent (%)	70	53	Min. 50
Water absorption (%)	–	1.2	Máx. 2

**Table 7:** Design parameters of the evaluated mixes.

Mix Type	Manufacturing Temperature	Total Bitumen Content	WTTF-Based Additive	WTTF-Based Additive	Commercial Cellulose Additive	Density	Stability	Flow	Air Voids	VMA	${VCA}_{MIX}$	${VCA}_{DRC}$	Binder Drainage
	(°C)	(% by Weight of Aggregate)	(% by Weight of AB)	(% by Weight of Aggregate)		(kg/m³)	(N)	0.25 mm	(%)	(%)	(%)	(%)	(%)
**HOT-MIX ASPHALT**													
HMA/R	154	5.3	0	-	-	2418	13,745	10.8	3.1	13.9	-	-	-
HMA/2A	154	5.3	2	-	-	2408	13,471	10.9	3.4	14.2	-	-	-
HMA/2B	154	5.3	2	-	-	2420	15,953	10.7	3.0	13.8	-	-	-
Chilean specifications for wearing course [47,50]							>9000	8–14	3–5	>13			
**STONE MASTIC ASPHALT**													
SMA/R	177	6.8	-	0	0.5	2333	13,087	12.1	4.2	18.2	31.3	40.1	0.12
SMA/0.5A	177	6.8	-	0.5	0	2328	13,232	14.1	4.2	18.4	31.2	40.1	0.17
SMA/0.5B	177	6.8	-	0.5	0	2327	13,232	13.5	4.3	18.4	31.2	40.1	0.16
Chilean specifications for wearing course [49]									4	>17	${VCA}_{MIX}$ < ${VCA}_{DRC}$		Max 0.3

**Table 8:** Results of the parameters obtained in 4-point fatigue test for HMA mixtures.

Mix Type	Initial Strain	Initial Phase Angle	Desv.	Final Phase Angle	Desv.	Dissipated Energy	Desv.
	(Microstrain)	(δ)		(δ)		(J/m^3^)	
HMA/R	300	38.0	1.5	43.8	1.9	11.1	5.5
	190	34.8	1.4	40.9	0.9	37.4	13.5
	150	33.1	0.9	39.7	2.0	75.6	11.7
HMA/2A	300	37.4	1.8	44.9	0.7	19.1	6.1
	190	34.4	1.7	40.8	0.3	66.6	12.1
	150	32.9	1.7	39.6	2.4	95.7	19.2
HMA/2B	300	37.3	1.7	45.7	1.6	20.2	4.6
	190	35.8	2.5	43.2	1.2	82.3	17.9
	150	34.7	1.6	42.4	1.6	111.6	28.6

**Table 9:** Results of the parameters obtained in the 4-point fatigue test for SMA mixtures.

Mix Type	Initial Strain	Initial Phase Angle	Desv.	Final Phase Angle	Desv.	Dissipated Energy	Desv.
	(Microstrain)	(δ)		(δ)		(J/m³)	
SMA/R	700	50.2	1.0	56.8	0.2	39.5	10.0
	500	48.6	1.9	54.6	1.4	120.2	69.8
	400	47.1	0.9	49.6	2.9	170.1	153.9
SMA/0.5A	700	49.7	2.0	56.7	0.9	51.6	18.5
	500	46.6	0.7	54.7	0.4	82.8	33.0
	400	47.0	1.7	52.3	1.3	289.8	279.9
SMA/0.5B	700	49.4	1.9	57.1	1.4	51.7	26.7
	500	46.2	1.0	53.5	1.3	100.8	60.4
	400	48.0	1.1	52.5	1.6	218.5	108.1

**Table 10:** The ANOVA results for the effect of the variables on the fatigue performance of the mixtures.

Source	Adjusted Sum of Squares	Adjusted Mean Square	F-Value	*p*-Value
Hot-mix asphalt				
Durability of asphalt mixtures				
Thickness (cm)	4.23876 × 10^16^	1.69551 × 10^15^	8.69	0.000
WTTF-based additive	2.14583 × 10^15^	1.07292 × 10^15^	5.50	0.000
Axle load	7.28573 × 10^15^	7.28573 × 10^15^	37.33	0.005
Stone mastic asphalt				
Durability of asphalt mixtures				
Thickness (cm)	8.23432 × 10^18^	3.29373 × 10^17^	8.17	0.000
WTTF-based additive	5.08331 × 10^17^	2.54166 × 10^17^	6.31	0.002
Axle load	1.59942 × 10^18^	1.59942 × 10^18^	39.70	0.000

## Data Availability

Data are contained within the article.
